# The power of desktop scanning electron microscopy with elemental analysis for analyzing urinary stones

**DOI:** 10.1007/s00240-023-01424-8

**Published:** 2023-03-15

**Authors:** A. Costa-Bauzá, F. Grases, F. Julià

**Affiliations:** Laboratory of Renal Lithiasis Research, University Institute of Health Science Research (IUNICS-IdISBa), University of Balearic Islands, Ctra. de Valldemossa Km 7.5, 07122 Palma, Spain

**Keywords:** Kidney stones, Morphocompositional study, Scanning electron microscopy, Stereoscopic microscopy, Infrared spectroscopy

## Abstract

The aim of this paper is to present a protocol for the routine morphocompositional study of kidney stones in a clinical setting, and to demonstrate that it is a simple and useful approach that can reliably determine the etiology of all types of kidney stones. Our routine study of kidney stones consists of a combination of stereoscopic microscopy, scanning electron microscopy, and infrared spectroscopy. The usefulness of such a procedure is demonstrated by its application to several illustrating examples. The protocol applied here is reliable and fast, and does not require multiple infrared spectroscopic analyses for most non-homogeneous samples. It also provides the identification of components that are present in very small proportions, the characteristics of internal and external structures, and information about areas with biological structures, such as renal tubules. It should be noted that results are obtained in a relatively short time and with high reliability. The detailed morphocompositional study of a urinary calculus is essential for establishing the diagnosis and etiology and for initiating the treatment of a patient with renal lithiasis, because there is a relationship between the specific characteristics of a stone and the specific etiology of the disease. The increasing number of treatments available for patients with different types of renal calculi makes improvements in diagnosis and determination of stone etiology, such as the procedure described here, more important now than ever.

## Introduction

The morphology and chemical composition of a kidney stone provides important information regarding the cause of its formation [1–3]. In fact, these are the most important data regarding stone formation, because the results of urinary analyses performed at the time of stone expulsion do not necessarily indicate the urinary conditions during its formation.

Morphocompositional studies of kidney stones [3–5] provide important clinical information about the:

- presence or absence of a region of attachment to the renal papilla;
- major component or components, which may be related to urine composition at the time of stone formation;
- minor components, especially relevant if they are located at the core of the stone [6–8];
- changes in composition over time (e.g., a layered structure with different components), which may be due to changes in seasons, diet, drug use, and lifestyle;
- changes in stone composition [calcium oxalate dihydrate (COD) transformation to calcium oxalate monohydrate (COM)] due to prolonged contact with urine while inside the urinary system [9, 10].

Some researchers have questioned the importance of morphocompositional studies of kidney stones, because the results are sometimes inconsistent with the results of biochemical analysis of 24-h urine [11]. However, this inconsistency actually confers more value to the results of morphocompositional studies, because they provide the best data about the alterations responsible for kidney stone genesis [1– 5].

Thus, morphocompositional study of urinary stones is essential to select complementary diagnostic tests and to determine the initial treatment for a patient suffering from kidney stones. In particular, the composition and structure of a kidney stone provides fundamental information regarding the specific pathogenesis of the disease and the underlying metabolic abnormalities. The need for a detailed study and analysis of kidney stones has increased in recent years because of progress in the specific treatments used for the different types of urolithiasis [12–20], and a better understanding of the risk factors for each type of renal calculus [1–5].

Research on kidney stones relies on many different techniques, from simple observations using stereoscopic microscopy (StM) to sophisticated techniques, such as micro-computed tomography (micro-CT), scanning electron microscopy with energy-dispersive X-ray microanalysis (SEM–EDS), and others [21–25]. However, the most common methods in clinical laboratories are wet chemical analysis and infrared (IR) spectroscopy. The advantages of wet chemical analysis are that it is relatively easy and inexpensive, and commercial kits are widely available. However, this method does not provide complete chemical information about the kidney stone, the results can sometimes be ambiguous, and the results are correct only about 50% of the time when examining samples with a single component [21, 26]. For this reason, experts in this field agree that classification of stones using wet chemical analysis is insufficient.

Another consideration is that most clinical laboratories routinely perform a qualitative or semi-quantitative analysis of kidney stones by IR spectroscopy of a pulverized part of the stone. This method is limited, because it does not provide information about minor components (< 5% of the stone) or the internal structure of the stone. Although information obtained by IR spectroscopy is valuable in providing general therapeutic advice, by itself, it does not provide sufficiently complete information about a renal calculus and its etiology [1, 3, 5]. For these reasons, it is currently accepted that no single method alone provides complete information regarding the structure and composition of a kidney stone, and that a combination of several techniques, including the traditional methods of StM and IR spectroscopy, is necessary to obtain complete, accurate, and clinically relevant information [3, 23, 27].

The objective of this article is to present a protocol for the routine study of kidney stones in a clinical setting, and to demonstrate that the combination of StM, SEM, and IR spectroscopy is a simple and useful approach that can reliably determine the etiology of all types of kidney stones.

## Recommended procedure for study of kidney stones

The external and internal composition and the morphology of a renal stone provide information about the urine composition during the period of stone development, and about other etiological factors related to the pathology, such as renal cavities and other anatomical conditions that favor urinary stasis [3, 5, 28]. Routine analysis must consider all these factors to provide all the information needed to guide clinical interventions.

The techniques used to study solid samples do not require dissolution, because it is important to know the species or substances that constitute the solid phase (oxalate, phosphate, calcium, magnesium, carbonate, etc.) and the specific nature of the solid phase. For example, calcium phosphates can precipitate in more than four different phases, and each is formed under certain conditions (pH, supersaturation, hydrodynamics, etc.). Knowledge of the solid phase present in the renal stone provides information about the possible alterations that led to its formation. On the other hand, as mentioned above, it is also very important to determine the global crystalline microstructure of a stone, because this also provides information about the timing and causes of stone formation.

Thus, to evaluate and study a kidney stone [2, 3, 4], it is essential to establish the:

- specific or general region of origin;
- presence of different crystalline phases and the sequence of their development;
- relationship with renal morphoanatomy and urine composition.

**Fig. 1:**
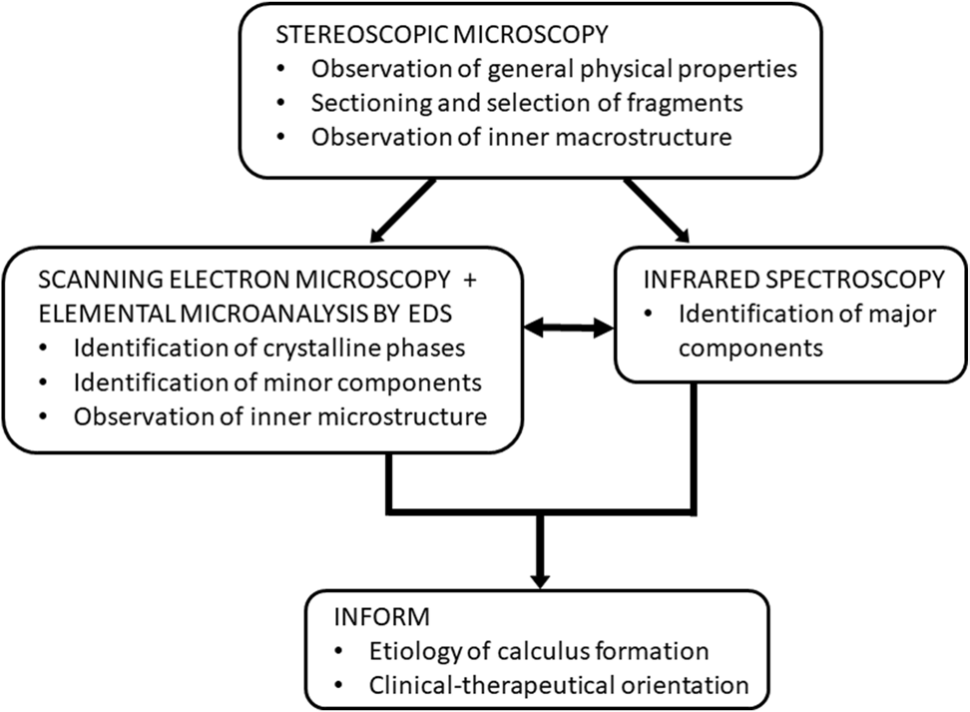
Recommended procedures to be used for the study of kidney stones

This information can only be obtained by a detailed morphocompositional study of kidney stones. The recommended procedures for the routine analysis of kidney stones therefore consists of a combination of observations using the conventional techniques (StM) and physical techniques, such as SEM–EDS and IR spectroscopy, as described in detail below and in Fig. 1.

The study of a renal calculus begins with direct observations of its external appearance using StM. Then, the stone should be divided into two parts, along a plane as close as possible to the geometric center, to allow analysis of the internal structure. In most cases, the results from this step will indicate the most appropriate procedures to be used in subsequent analysis.

A detailed study of the internal structure of the calculus and the detection and identification of microcomponents requires the use of SEM–EDS. Analysis by IR spectroscopy of one or several parts of the stone is necessary if the results from SEM–EDS do not allow unequivocal establishment of its composition. If SEM–EDS is not available, then IR spectra of various zones of the stone should be performed, including the initial or core zone, the intermediate zone, the superficial layer, and any zone that differs in appearance from the rest of the stone. Obviously, this approach has several drawbacks, in that it can be time-consuming, it cannot detect a component that accounts for less than 5% of the stone when its presence is not detected by StM, and the procedures lead to sample destruction.

It should be noted that in all cases, personnel dedicated to the study of renal stones must have solid training and experience in the study of renal stones and the analytical techniques, so that significant and reliable data related to etiology can be determined. This professional experience is especially important when the stones to be studied are fragmented due to the surgical treatment needed for removal. In particular, study of the relationship of different types of stone fragments with calculus composition requires a great deal of experience in the study of whole calculi.

## Review and discussion of techniques

Below, we provide a brief discussion of the major types of results that can be obtained using these three techniques for the routine study of kidney stones.

## Stereoscopic microscopy

Most researchers agree that the first step in the analysis of a stone should be examination by StM [2, 3, 22, 27]. StM provides enlarged images of samples of various sizes using visible light, and there is no need for sample preparation or destruction. The magnification typically ranges from 4 × to 100 × , the instruments are very affordable, and most instruments have cameras.

In practice, the observer first uses StM to examine the external surface of the intact stone to determine its shape, dimensions, color, evidence of stone contact with the kidney epithelium, presence of superficial deposits, and other gross characteristics. This external characterization allows the formation of some hypotheses about the composition and possible structural arrangement of the stone and about the characteristics of the kidneys and urine. For example, the presence of certain surface deposits that differ in composition from the bulk of the stone may indicate the stone grew due to changes in the chemical composition of the urine. This can occur in some calcium oxalate monohydrate (COM) stones, which may present with superficial deposits of calcium oxalate dihydrate (COD) crystals.

In addition, an external examination provides important information about optimal fracture plane to be used for obtaining the information about the internal structure of the stone. The sectioning of a lithiasic sample must be performed carefully to prevent alterations or destruction of any structure of interest.

Many of the major components of a kidney stone can be reliably identified when sections are examined by StM. For example, examination of stone sections enables the observer to distinguish calcium oxalate from calcium phosphate. Even more importantly, the inspection of stone sections using StM provides information about the structural characteristics of a stone, such as the degree of internal organization, the location and size of the stone core, the presence of layers and/or a radial structure, the order of deposition of the different components, and additional details. If the calculus is already fragmented, then all fragments should be carefully observed to identify as many structural and compositional details as possible.

In conclusion, StM can be used to observe the exterior and interior of a calculus, and provides information that allows the selection of regions of the sample that are most important for analysis by other techniques. StM examination also provides information related to the history of the stone and insights regarding the cause or causes of stone formation [3, 4, 5, 29, 30, 31].

This information includes the:

- external morphology and color;
- presence or absence of crystalline or amorphous surface deposits;
- presence or absence of a point of attachment to a renal papilla;
- optimal fracture plane/s to be used to information about the internal structure (in non-fragmented stones);
- presence or absence of one or more cores;
- internal structure, which may be organized (with a radial or layered structural pattern), disordered (no crystalline structures), or a mixture of both;
- areas of the stone with different composition based on color, texture, and crystalline morphology;
- order of deposition of the different stone components.

On many occasions, even when StM can identify the presence of cores or layers of different composition, it does not allow the identification of the different phases that make up the stone, making it necessary to use other techniques. Nonetheless, the results from StM provide important guidance regarding the most appropriate steps needed for subsequent study. In these cases, identification can be made by SEM–EDS (which provides detailed elemental analysis of selected areas) and IR spectroscopy (although this technique cannot identify compounds that account for less than 5% of the analyzed portion [32]).

## Scanning electron microscopy

SEM, like StM, is a non-destructive technique that provides observations of the structure of a kidney stone, although SEM can provide magnifications of 20,000 × or more. In addition, the images from SEM obtained in the backscattered electron mode provide information about the three-dimensional structure of a stone and a very clear characterization of crystal morphology.

An important consideration is that a substance present in minute quantities that is not detectable by IR spectroscopy can be decisive in establishing stone etiology. To determine the importance of these microcomponents, it is necessary to have precise knowledge of the fine structure of the calculus. SEM can help to provide information regarding the initial area of calculus development and a key to the origin of the calculus.

The methodology currently used for SEM consists of placing the stone on a sample holder and fixation with adhesive conductive tape, with no need to cover the sample with gold. After observation and analysis, the sample is in the same state as before SEM analysis. Furthermore, SEM can be used with auxiliary techniques, especially X-ray scattering analysis (EDS, Energy Dispersive Spectrometry). This provides great value in the study of stone microstructure and analysis of the minor components of kidney stones [33–37]. EDS can provide reliable data on the elemental composition of a specific point or general area of a stone. Although previous EDX models could not detect the first eight elements [22], technical advances of certain detector models, such as the one used in this study, have allowed the detection of elements from atomic number 6, that is, C, N and O, important elements in the case of uric acid and ammonium urate or to establish the degree of carbonation of apatites. Even though it is not yet possible to use EDS to detect the four elements with the lowest molecular weights (H, He, Li, and Be), this does not significantly impair its ability to identify major stone components (Table 1).

**Table 1:** Elements detectable by energy-dispersive X-ray (EDS) analysis of kidney stones

Kidney stone component	Formula	Elements*	Molar percentage**
Calcium oxalate monohydrate	CaC_2_O_4_.H_2_O	C O **Ca**	25% 62.5% 12.5%
Calcium oxalate dihydrate	CaC_2_O_4_.2H_2_O	C O **Ca**	22% 67% 11%
Brushite	CaHPO_4_.2H_2_O	O **P Ca**	75% 12.5% 12.5%
Apatite	Ca_10_(PO_4_)_6_(OH)_2_	O **P Ca**	62% 14% 24%
Carbapatite	Ca_10_(PO_4_)_6_(OH)_2-x_(CO_3_)_x_	C O **P Ca**	2% 62% 13% 22%
Magnesium ammonium phosphate	MgNH_4_PO_4_.6H_2_O	N O **Mg P**	8% 77% 8% 8%
Uric acid (anhydrous or dihydrate)	C_5_H_4_N_4_O_3_	C **N** O	42% 33% 25%
Monosodium urate monohydrate	NaC_5_H_3_N_4_O_3_.H_2_O	C **N** O **Na**	36% 29% 29% 7%
Potassium urate	KC_5_H_3_N_4_O_3_	C **N** O **K**	38% 31% 23% 8%
Ammonium urate	NH_4_C_5_H_3_N_4_O_3_	C **N** O	39% 39% 22%
Calcium urate	Ca(C_5_H_3_N_4_O_3_)_2_.6H_2_O	C **N** O **Ca**	32% 26% 39% 3%
Cystine	C_6_H_12_N_2_O_4_S_2_	C N O **S**	43% 14% 29% 14%

^*^The principal element needed for identification is in bold

^**^The presence of several components in the same area affects the molar percentage, preventing the use for certain stones

Thus, SEM–EDS can provide information about the:

- morphology of crystals in the stone, and in many cases their unequivocal identification;
- identity and location of crystalline phases and minor components;
- identity of the starting point(s) of calculus formation;
- changes in the crystalline shape or composition, and their relationship to stone growth;
- internal structure, with confirmation of the StM observations.

As mentioned above, many renal calculi are already fragmented prior to analysis, and this represents a partial loss of information. However, an observer who has extensive experience about the structure of kidney stones can use the StM results to select several representative fragments, and then use SEM–EDS to provide additional information essential for determining stone etiology.

Therefore, together with StM and IR spectroscopy, SEM–EDS is a fundamental tool for the study of kidney stones, and the information it provides has great clinical and practical importance, justifying its routine use in clinical practice. SEM will be used more in the future due to the development of desktop models that are easy to use, more affordable, and provide results with the same quality as larger and more expensive models.

## Infrared spectroscopy

IR spectroscopy is a technique commonly used for the routine analysis of solids, such as kidney stones, that does not require sample dissolution [36–41]. IR spectroscopy is useful for identifying organic and inorganic compounds, and is particularly useful for determining the specific functional groups in a molecule, because these groups vibrate at nearly the same IR frequencies regardless of the molecular environment. IR spectroscopy provides a “fingerprint” that is unique and specific to each substance. The IR spectrum of a sample provides identification of its components, and has the additional advantage of identifying non-crystalline compounds and the degree of hydration. In addition, advances in computerized IR spectroscopy, particularly Fourier Transform IR (FTIR) spectroscopy, have made it possible to obtain IR spectra in less than 1 min. Finally, it should be noted that FTIR can be performed using a sample of less than 1 mg, although a disadvantage is that FTIR is a destructive technique.

The KBr pellet method, a specific technique recommended for this analysis, requires 1 mg or even less of a stone sample. For a solid sample, such as a kidney stone, preparation requires 0.4 to 1.0 mg of the sample to be dispersed in a KBr matrix and then pressed into a clear pellet.

For samples whose composition could not be determined by SEM–EDS, if the overall appearance is homogeneous, then it can be analyzed by IR spectroscopy. However, if the sample is heterogeneous and contains areas of different colors, textures, layers, or other structural details, then identification of the different phases must be performed by IR spectroscopy of each specific area, and samples must be carefully obtained from the different regions of the calculus using a stylet or scalpel. In addition, when a stone is drug-induced or has a very unusual composition, IR spectroscopy can be used to identify the individual components.

The IR spectrum of a sample fragment may correspond to a pure compound or to a mixture of several compounds. Up to 65 different molecules can occur in urinary stones, including several of exogenous origin, although only a small number of these molecules are common (Table 1). Thus, using a collection of IR spectra of different compounds makes possible to identify the component present in a stone, although this can be problematic when the calculus has a mixed composition. Finally, IR spectroscopy cannot identify compounds that account for less than 5% of the stone, and SEM–EDS must be used for analysis of these minor compounds.

## Results

We presented nine examples below that illustrate the application of the recommended procedures. It should be noted that the morphocompositional study of kidney stones described in Fig. 1 provides results in a relatively short time and with high reliability for most samples. The time required for this analysis combining StM and SEM is about 5 min, and will not exceed that dedicated to the study of pathological anatomy samples, for which detailed observation is also required, typically using transmission electron microscopy (TEM) [42].

For all examples below, an MOTIC SMZ-161 stereoscopic microscope (MoticEurope, Barcelona, Spain), a Hitachi TM4000 Plus II desktop scanning electron microscope (Hitachi, Tokyo, Japan) coupled with Quantax 75 EDS microanalyzer (Bruker, Berlin, Germany), and a Bruker Hyperion IR spectrometer (Bruker, Berlin, Germany) were used for analysis. All kidney stones were from the Health Service of the Autonomous Community of the Balearic Islands, and were analyzed immediately after collection.

### Example 1 (Fig. 2)

**Fig. 2:**
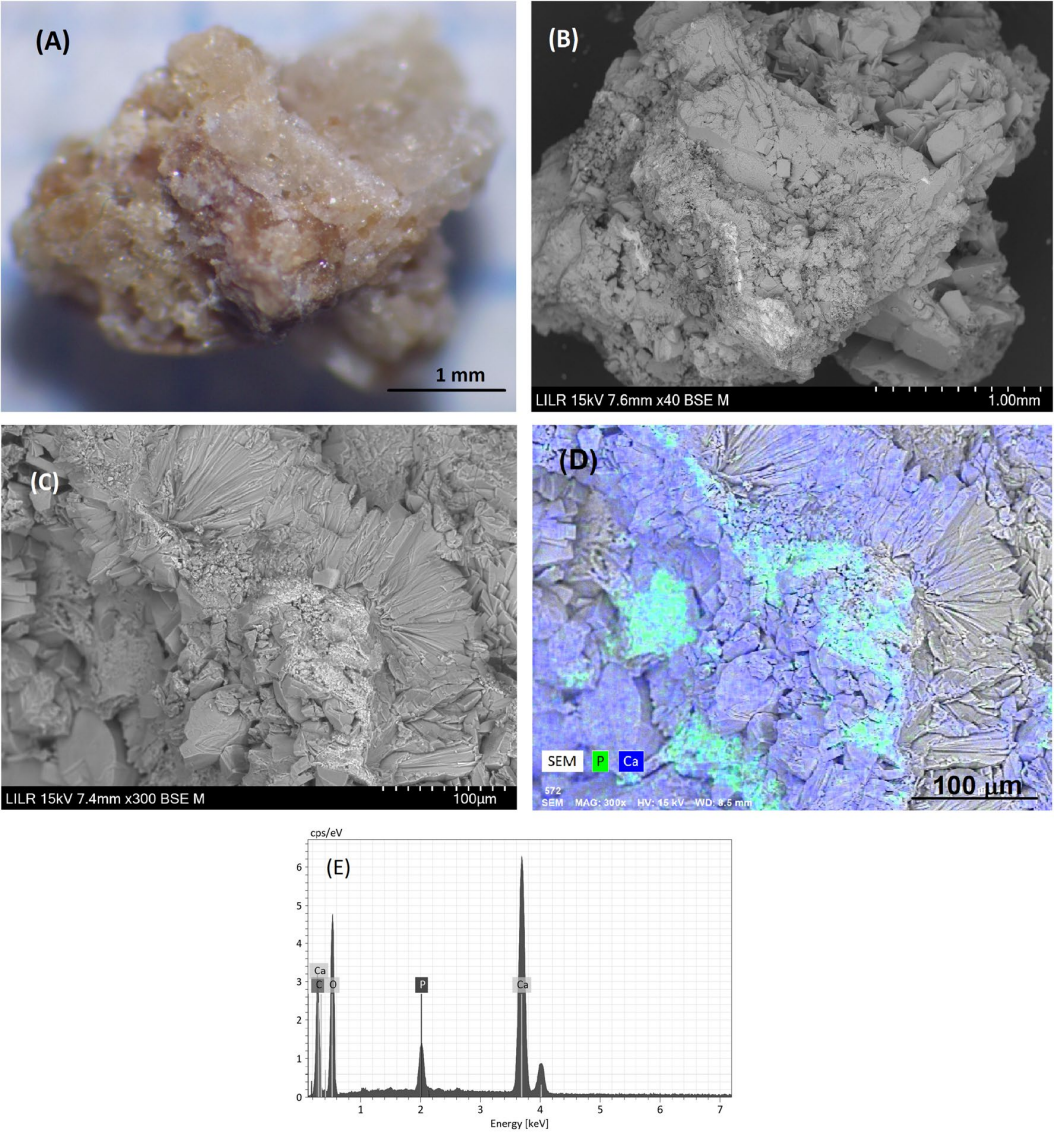
StM (**A**) and SEM (**B**) views of the whole renal stone. Details of the central region (**C**), its elemental distribution map (Ca: blue, P: green) (**D**), and its spectrum from EDS analysis (**E**)

This is a typical COD stone, a composition mainly associated with hypercalciuria. Although SEM–EDS clearly indicates the presence of a small quantity of apatite (AP), neither StM nor IR spectroscopy can detect AP. The presence of AP is important, because it indicates a urinary pH higher than 6.2, at least for some period of time. It is also necessary to consider that AP is a very effective heterogeneous nucleant of COD crystals [43, 44].

### Example 2 (Fig. 3)

**Fig. 3:**
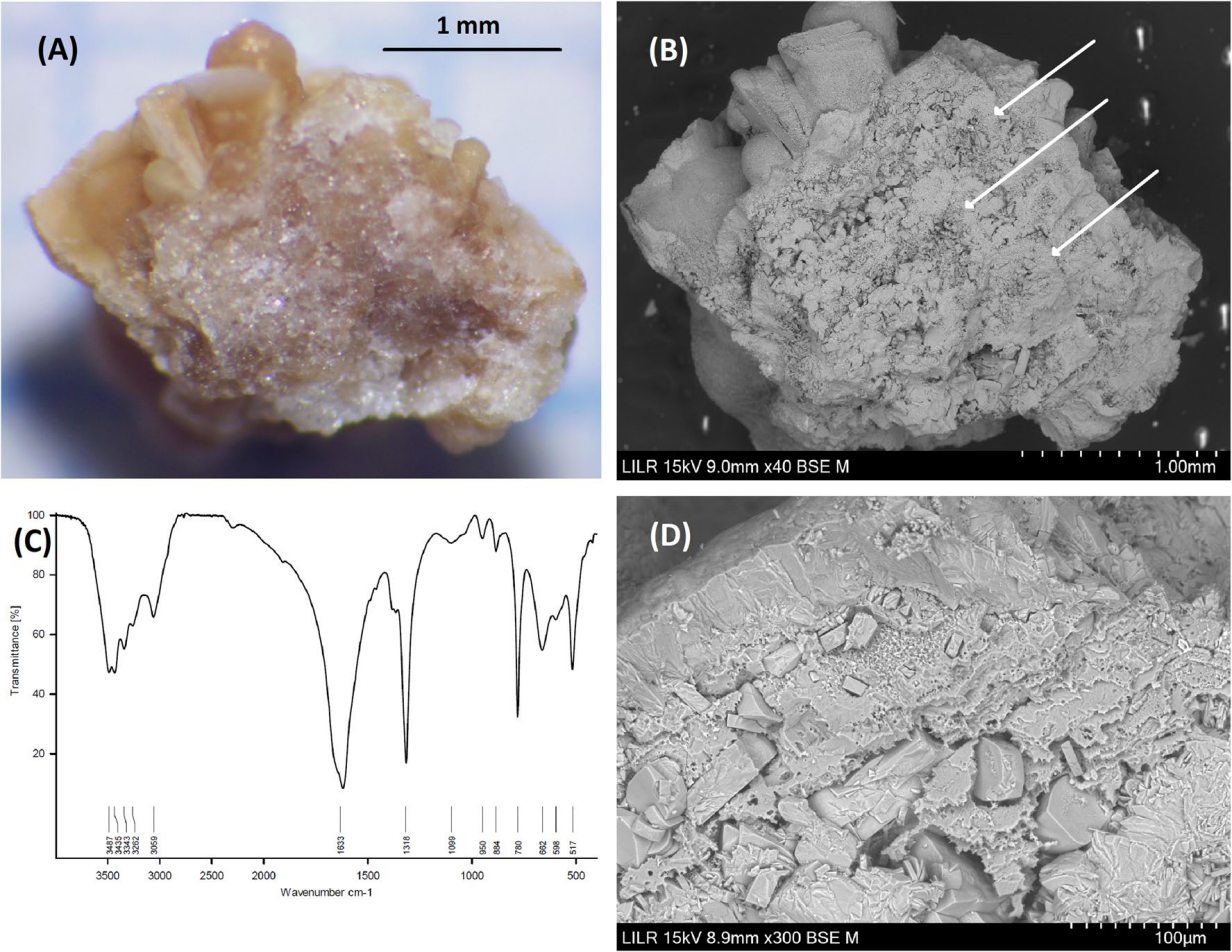
StM (**A**) and SEM (**B**) views of a section of the renal stone, with arrows indicating areas where the original COD crystals were enclosed. The IR spectrum matches the IR spectrum of COM (**C**). Detail of a zone in which large COM crystals are immersed in a mass that originally corresponded to a COD crystal

This is a calcium oxalate calculus that originally formed as COD, but transformed into COM due to urine-mediated processes. The IR spectrum clearly corresponds to COM. SEM observations of the internal structure show the typical large COM crystals (transformed from COD crystals), and some areas where the original COD crystals were enclosed (Fig. 3B, D). The structure of this stone indicates it was in the kidney and bathed in urine for a long time, and that stone formation occurred, because the patient had a high concentration of urinary calcium, probably hypercalciuria.

### Example 3 (Fig. 4)

**Fig. 4:**
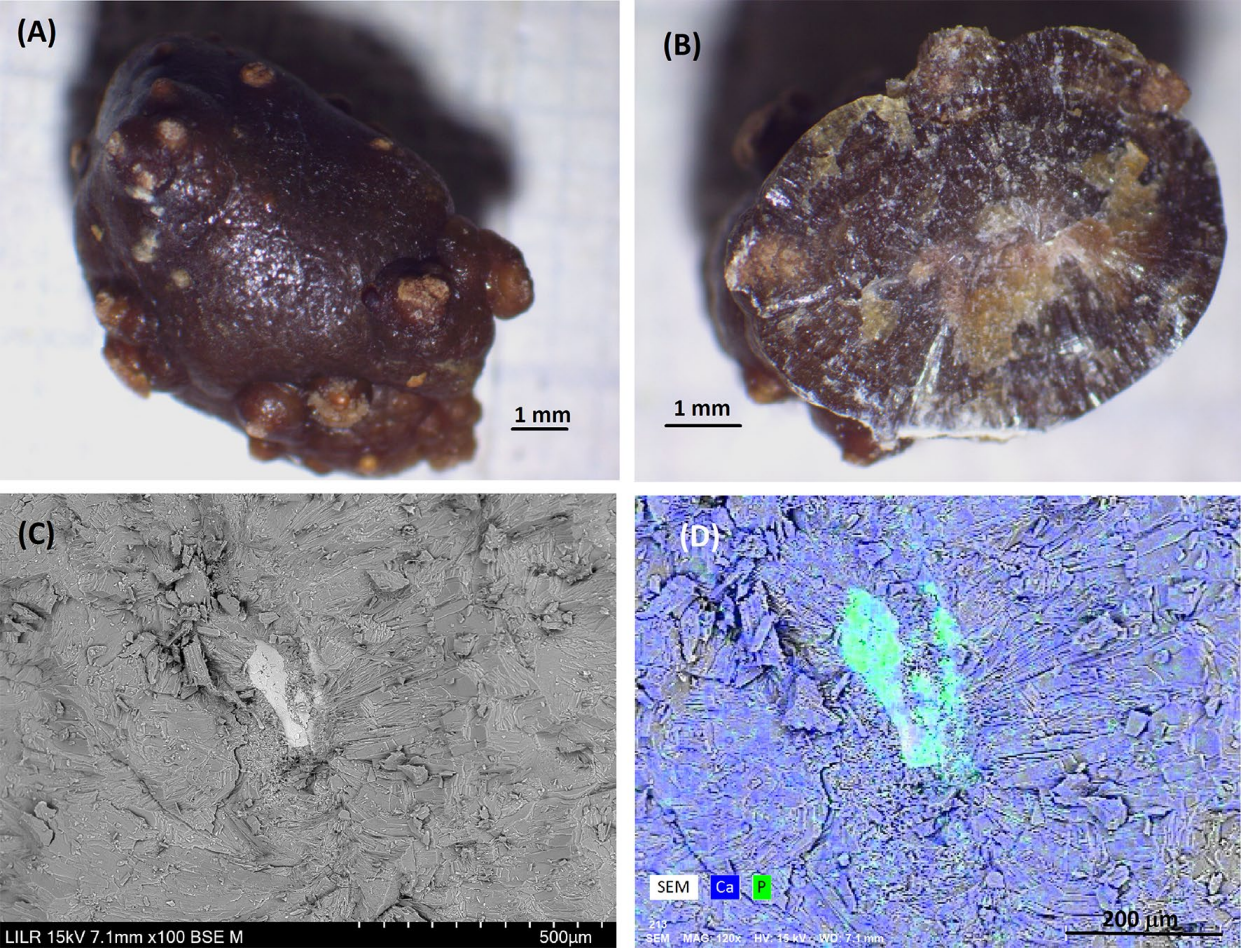
StM of the whole renal stone (**A**) and of a cross-section (**B**). SEM view of a zone located in the center of the stone (**C**) and its corresponding elemental distribution map obtained by EDS analysis (Ca: blue; P: green) (**D**)

This is a COM stone with spheroidal symmetry, but without points that attached to the epithelium, so it is not a papillary calculus (Fig. 4A and B). This stone probably developed in a cavity with low urodynamic efficiency. The cross-section indicates that development started from the central area, but StM does not provide identification of any component. However, SEM + EDS indicates the presence of AP (Fig. 4C and D). This may indicate the importance of the heterogeneous nucleation of calcium oxalate on the AP formed at a urinary pH above 6.2, and should be confirmed by urinalysis.

### Example 4 (Fig. 5)

**Fig. 5:**
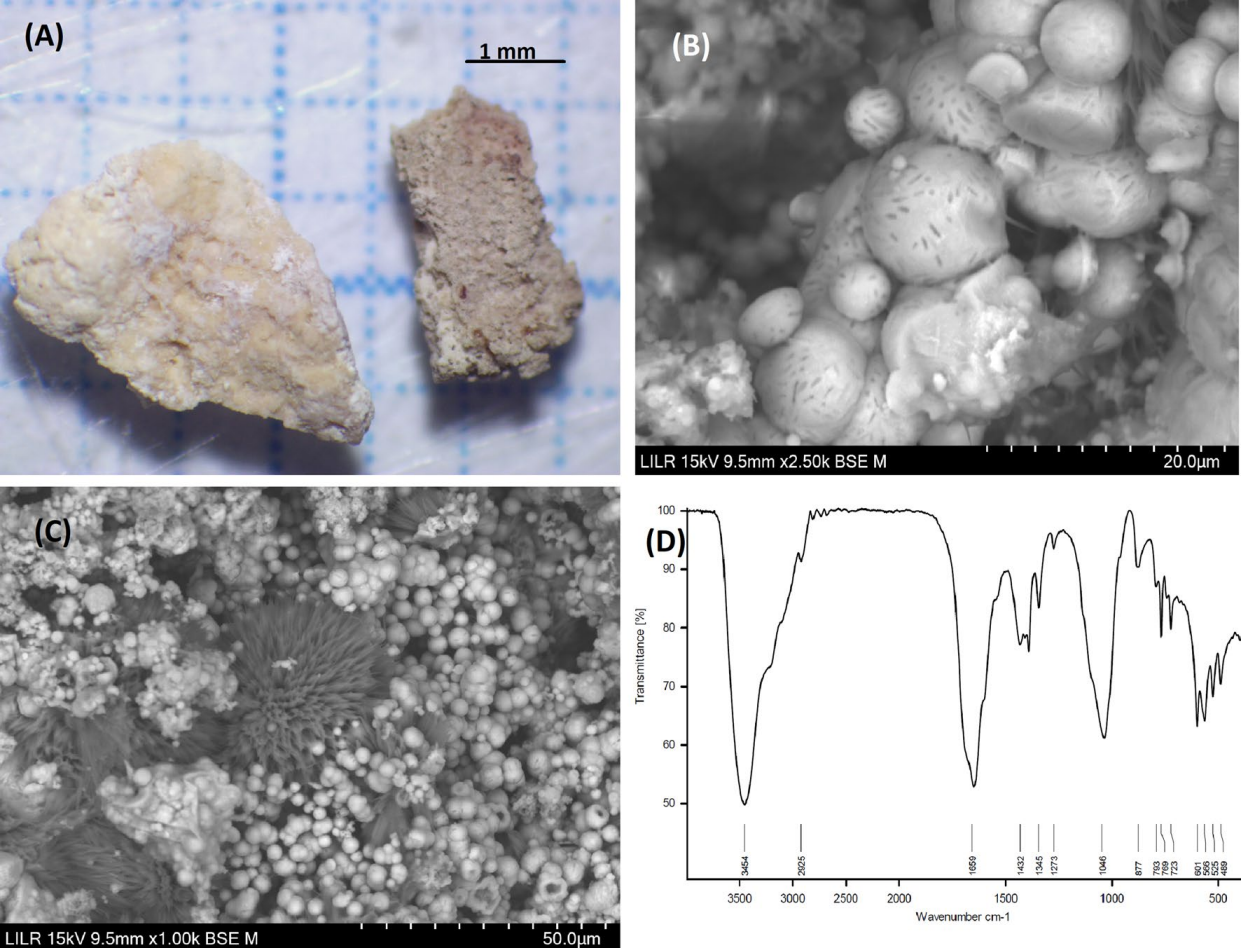
StM of the two fragments of the renal stone sample (**A**). SEM images of a zone in which AP spherulites with bacterial imprints are present (**B**) and another zone that has AP and spherulites of urate salts (**C**). IR spectrum of a fragment indicates AP with small amounts of urates (**D**)

This is an AP fragmented calculus in which SEM–EDS detects AP, organic matter, and a small number of urate spherulites, but without evidence of struvite. In addition, there is evidence of many bacterial imprints inside the stone (Fig. 5B). The absence of struvite indicates that these are non-ureolytic bacteria, probably *E. coli* or *Enterococcus*. These bacteria can proliferate at urinary pH values above 6, although these specific bacteria are not responsible for the high urinary pH. The presence of AP and urates demonstrates the stone formed at a urinary pH greater than 6.2. Verification of the species of bacteria is very important, so appropriate antibiotics can be administered to prevent complications [45].

### Example 5 (Fig. 6)

**Fig. 6:**
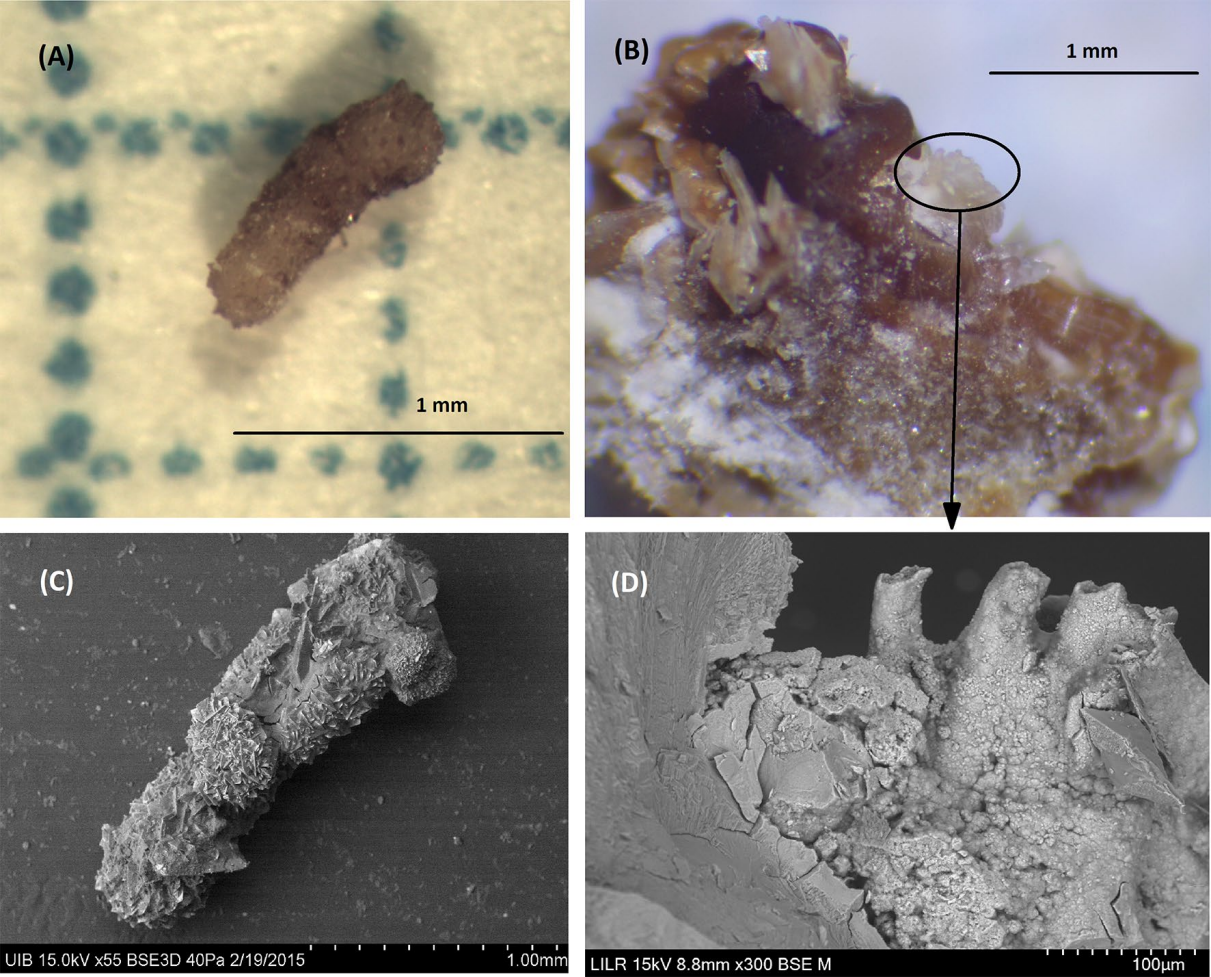
StM (**A**) and SEM (**C**) of a whole renal stone (Randall’s plug blocking Bellini’s duct) obtained by percutaneous nephrolithotomy (PNL). StM of a section of a papillary renal stone (**B**) and SEM details of the surrounded area, in which there are AP plugs that induced the formation of this calculus (**D**)

This are two cases of calcium oxalate calculi that were attached to the tip of a renal papilla. The formation of tubular plugs, due to high calciuria and urinary pH greater than 6.0, leads to deposits of COD + AP in the terminal regions of the tubules. These have contact with urine and lead to development of COD stones that remained attached to the renal papillary tip for at least a short time (Fig. 6A and C). SEM observations of these calculi enables to identify the area of attachment to the papilla (organic matter and calcified tubules). These patients usually develop many stones, and because of the papillary lesions, they sometimes also develop papillary COM stones (Fig. 6B and D).

### Example 6 (Fig. 7)

**Fig. 7:**
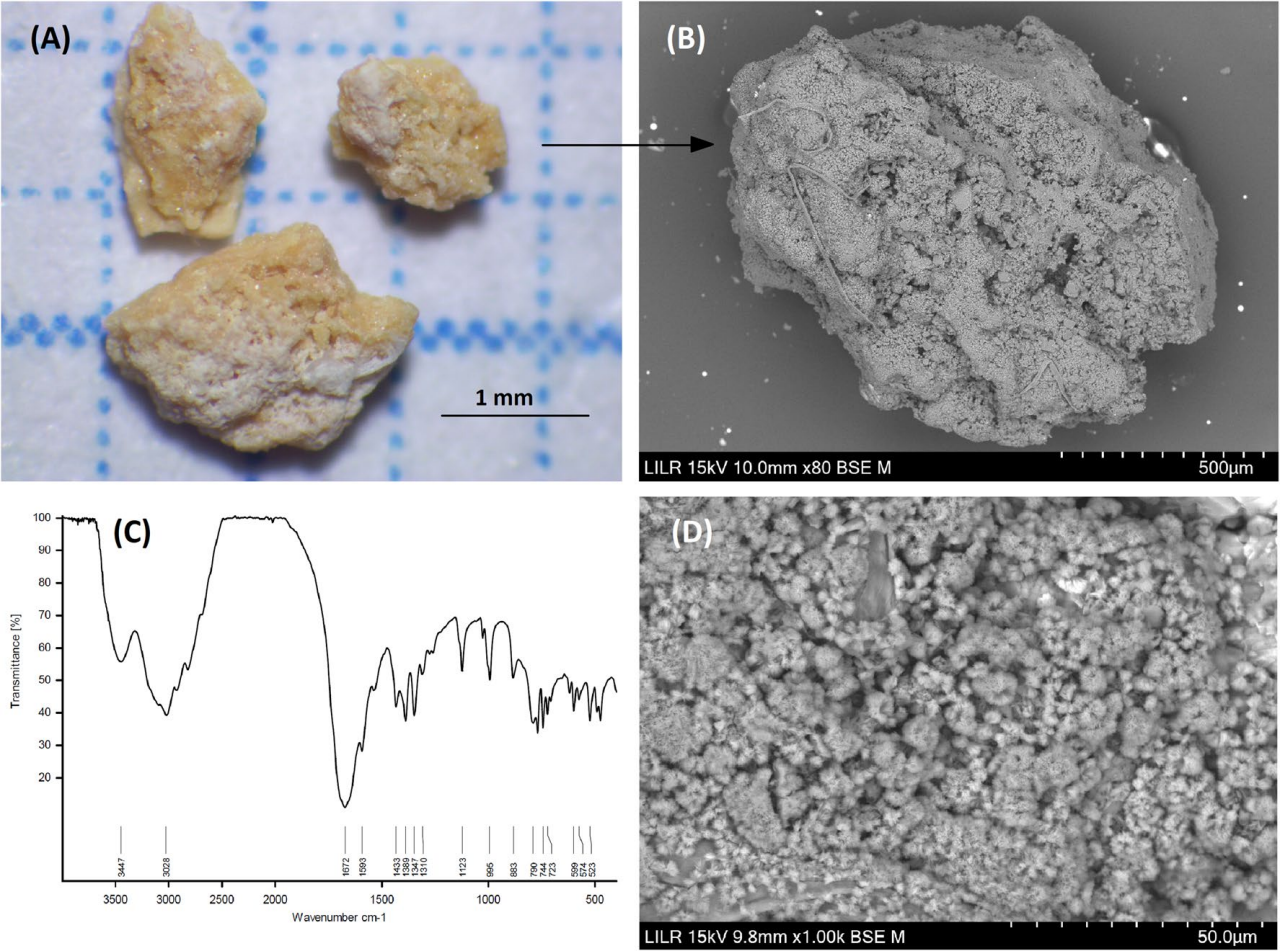
StM (**A**) and SEM (**B**) of the whole renal stone. An FTIR spectrum indicates uric acid dihydrate (**C**). Details of a zone in which small potassium and sodium urate spherulites covered the surface (**D**)

This is a uric acid stone that has small deposits of Na/K urate on the surface based on SEM–EDS (Fig. 7B and D). The formation of these deposits must be due to an increase in urinary pH over 6.5 values, likely associated with an alkalinization therapy of the urine. These deposits are not detectable by StM (Fig. 7A) or FTIR (Fig. 7C) due to small amount. These deposits point to the importance of effective pH control when a patient with uric acid stones modifies the diet or receives urine alkalizing therapy.

### Example 7 (Fig. 8)

**Fig. 8:**
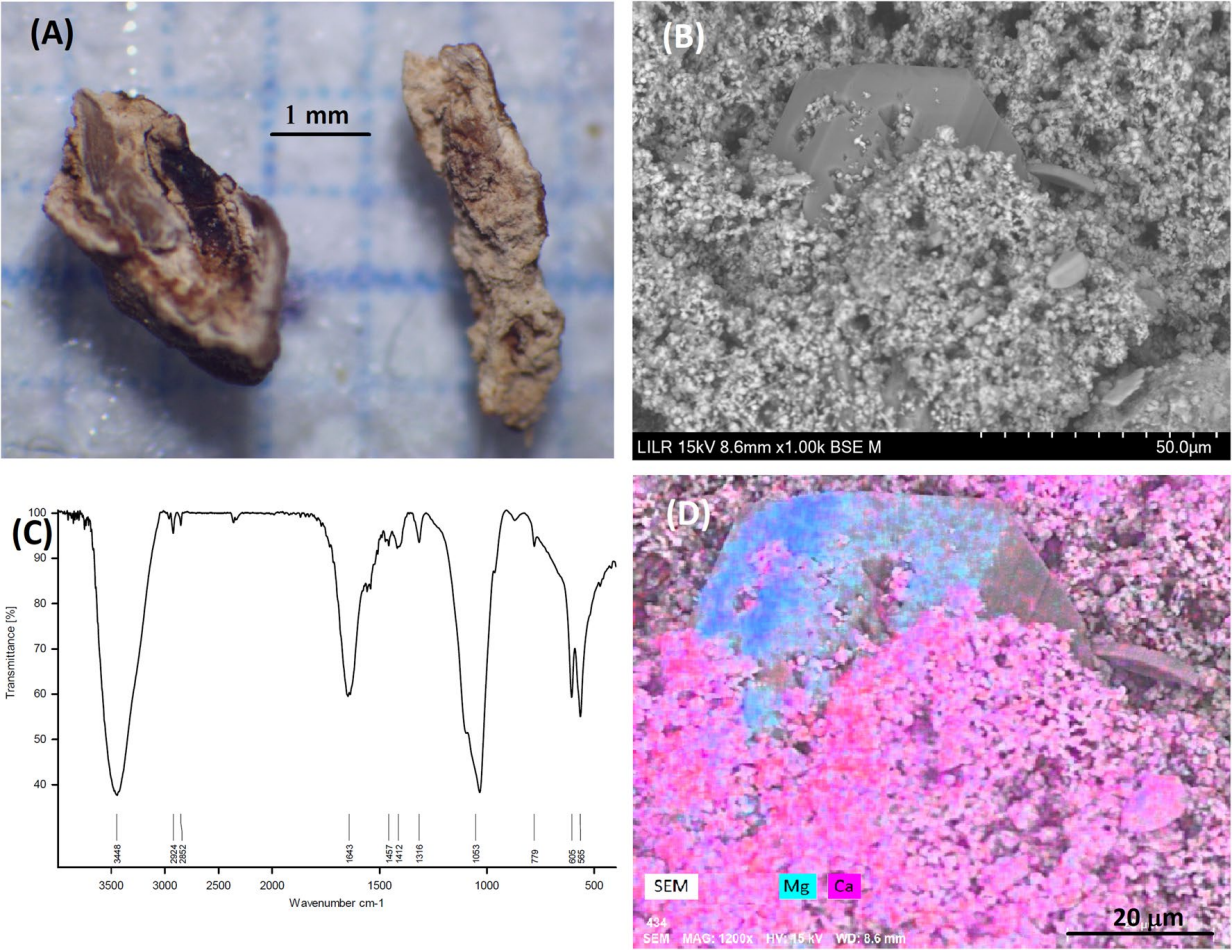
StM of two fragments of the renal stone sample (**A**). SEM of a portion of the stone showing a magnesium ammonium phosphate crystal (struvite) surrounded by AP (**B**). FTIR spectrum indicating apatite (**C**). Elemental distribution map obtained by EDS (Ca, pink; Mg, teal) (**D**)

This is an AP calculus with small amounts of COM and magnesium ammonium phosphate (detected by SEM + EDS), indicating a urinary infection. Due to the low amount of struvite, it was undetectable by StM or FTIR spectroscopy (Fig. 8C). However, it is necessary to verify the presence of struvite to confirm the infectious origin of the stone.

### Example 8 (Fig. 9)

**Fig. 9:**
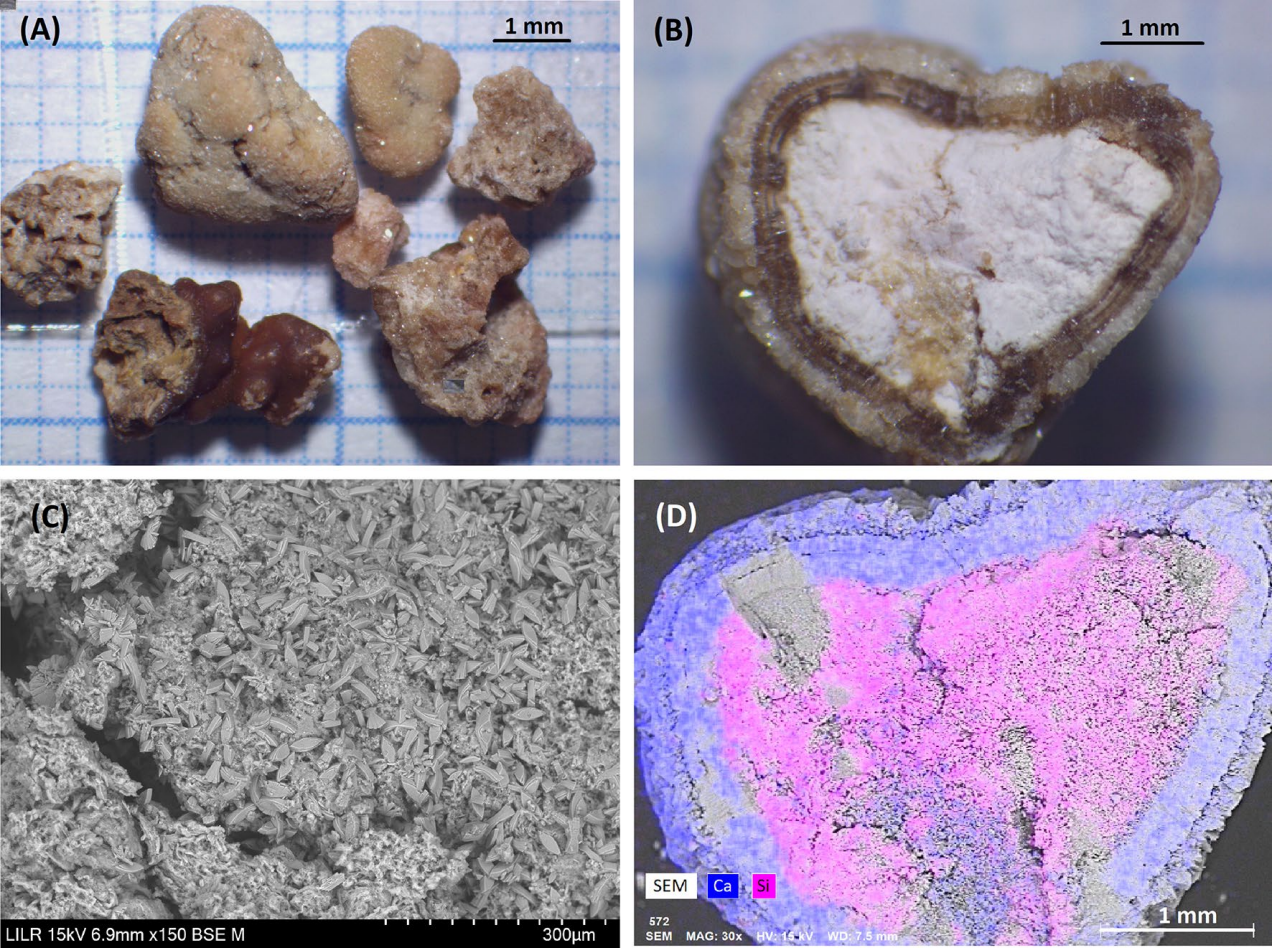
StM of the whole sample (**A**) and cross-section of one stone (**B**). SEM of a zone in the central region, in which small COM crystals are present (**C**). Elemental distribution map of the section obtained by EDS analysis, in which Ca (blue) is in the external layer and Si (pink) is in the central region

These are samples of small calculi that had a thin layer of COD on the outside (Fig. 9A). A cross-section shows the presence of another layer of COM (Fig. 9B). The central part of the calculus, which formed first and accounts for the largest part, is a mass of silica (Fig. 9B and D), in which many small individual crystals of COM are present (Fig. 9C). The structure of this stone is completely atypical, and it is therefore necessary to determine the reason for silica in the urine of this patient, which may be related to its origin from West African countries, where clay consumption has been suggested to be the reason for these types of kidney stones [46].

### Example 9 (Fig. 10)

**Fig. 10:**
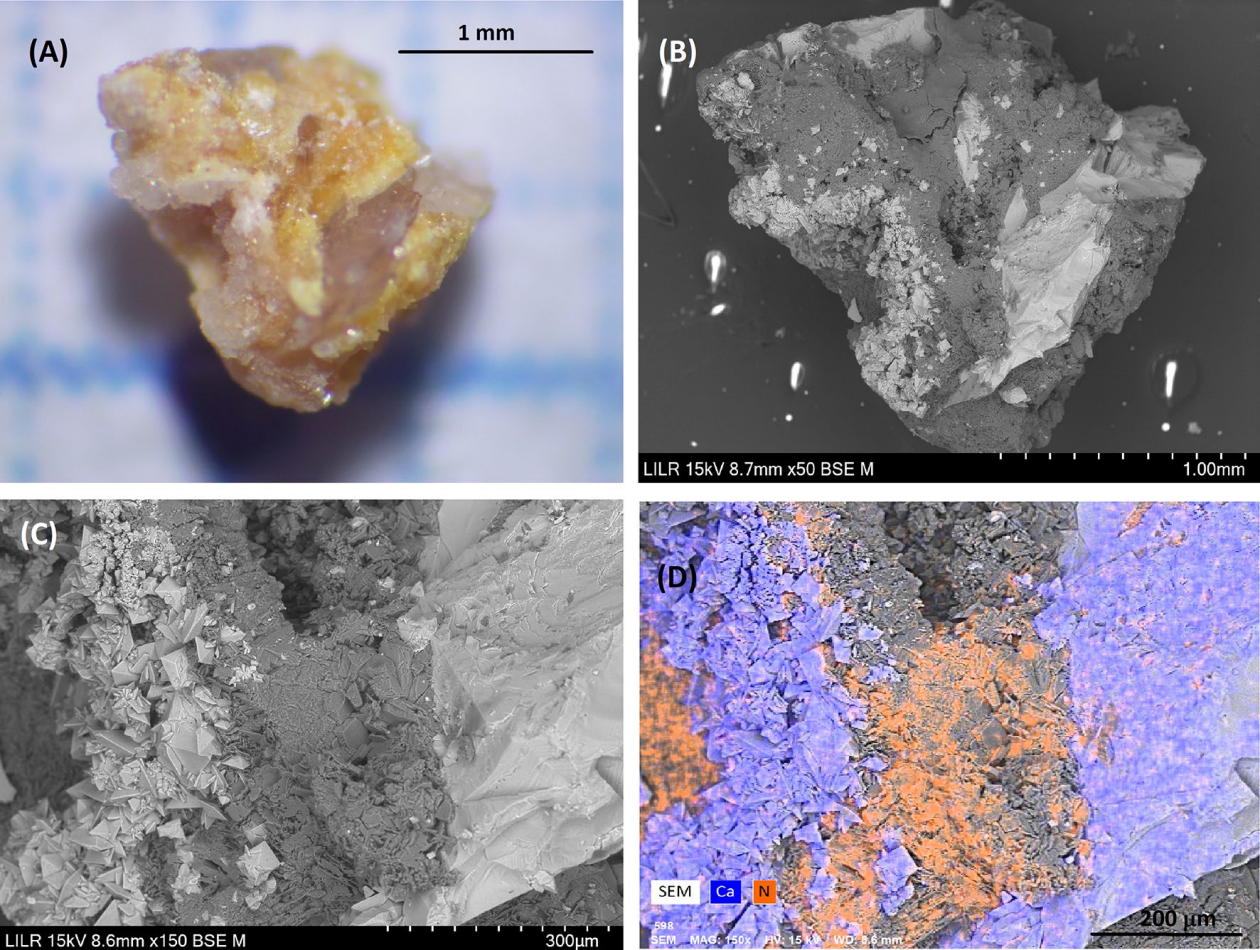
StM (**A**) and SEM (**B**) of the whole renal stone. A detail of a zone in a central part (**C**) and its corresponding elemental distribution map from EDS analysis (**D**) shows the presence of Ca (blue) and N (orange)

This is a mixed uric acid/COD stone, which only rarely co-occurs in kidney stones. StM observations led to the suspicion of these two components (Fig. 10A), and SEM–EDS confirmed this (Fig. 10C and D). IR spectroscopy of the sample was not necessary.

## Conclusions

The detailed analysis of a urinary calculus is essential for establishing the diagnosis and etiology and for initiating the treatment of a patient with renal lithiasis, because there is a relationship between the specific characteristics of a stone and the specific etiology of the disease [1–5]. The major components of a kidney stone (COM, COD, uric acid, calcium phosphates, magnesium ammonium phosphate, cystine) can be obtained directly by IR spectroscopy (the most commonly used analytical method), but IR spectroscopy data alone are no longer sufficient to determine the etiology of different types of renal stones. A detailed structural study and an analysis of the major and minor components are needed to provide sufficient information to determine the possible causes of stone formation. Detailed studies of a renal stone should be presented to the clinician prior to initiation of treatment. The detailed study of renal stones should use StM, SEM–EDS, and often IR spectroscopy (Fig. 1, Table 2).

**Table 2:** Information about renal calculi provided by StM, SEM–EDS, and IR spectroscopy

Information	StM	SEM–EDS	IR spectroscopy
Initiation site (central or papillary anchor)	Yes	yes	No
Overt characteristics (color, shape, internal structure)	Yes	yes (no color)	No
Surface deposits	Yes	Yes	No
Major components (crystalline morphology)	In many cases	Yes	Yes
Zones with different composition and their order of formation	In most cases	Yes	No
Minor components	No	Yes	Only if > 5%
Internal microstructure	No	Yes	No
Infrequent components, such as organic drugs	No	No	Yes (requires spectrum library)

The techniques used for the morphocompositional study of renal calculi described here are reliable and fast, and do not require multiple IR spectroscopic analyses for most non-homogeneous samples. Our recommended procedures also provide the identification of components that are present in very small proportions (undetectable by FTIR), the characteristics of internal and external structures, and information about areas with biological structures, such as renal tubules. The combined use of StM and IR spectroscopy is a useful choice, although this alternative approach requires more time and does not provide information on minor components.

It must be remembered that renal lithiasis is a multifactorial disease. Therefore, precise knowledge of the factors that affect the development of the different types of kidney stones is important, and only a thorough study of a kidney stone can provide sufficient information to establish its etiology. Urinalysis is necessary, because it can provide evidence suggestive of stone etiology; however, urine chemistry changes over time, and the absence of certain alterations at the time of urinalysis is not a reason of dismiss them as etiological factors, if the study of the renal calculus, performed with the methods described here, indicates their role in its development.

Finally, it should be considered that there are new treatments available for patients with different types of renal calculi, as phytate for calcium stone formers [12–15], theobromine for uric acid stone formers [16–19], or methionine for phosphate stone formers [20] in addition to those used so far. Thus, improvements in diagnosis and determination of stone etiology that use the procedures described here are more important now than ever.

## Data Availability

Data sharing not applicable to this article as no datasets were generated or analysed during the current study.
